# β-Lapachone Significantly Increases the Effect of Ionizing Radiation to Cause Mitochondrial Apoptosis via JNK Activation in Cancer Cells

**DOI:** 10.1371/journal.pone.0025976

**Published:** 2011-10-06

**Authors:** Moon-Taek Park, Min-Jeong Song, Hyemi Lee, Eun-Taex Oh, Bo-Hwa Choi, Seong-Yun Jeong, Eun-Kyung Choi, Heon Joo Park

**Affiliations:** 1 Department of Microbiology, Center for Advanced Medical Education by BK21 Project, College of Medicine, Inha University, Incheon, Republic of Korea; 2 Department of Radiation Oncology, Asan Medical Center, College of Medicine, University of Ulsan, Seoul, Republic of Korea; Roswell Park Cancer Institute, United States of America

## Abstract

**Background:**

β-lapachone (β-lap), has been known to cause NQO1-dependnet death in cancer cells and sensitize cancer cells to ionizing radiation (IR). We investigated the mechanisms underlying the radiosensitization caused by β-lap.

**Methodology/Principal Findings:**

β-lap enhanced the effect of IR to cause clonogenic cells in NQO1^+^-MDA-MB-231 cells but not in NQO1^−^-MDA-MB-231 cells. β-lap caused apoptosis only in NQO1^+^ cells and not in NQO1^−^ cells and it markedly increased IR-induced apoptosis only in NQO1^+^ cells. Combined treatment of NQO1^+^ cells induced ROS generation, triggered ER stress and stimulated activation of ERK and JNK. Inhibition of ROS generation by NAC effectively attenuated the activation of ERK and JNK, induction of ER stress, and subsequent apoptosis. Importantly, inhibition of ERK abolished ROS generation and ER stress, whereas inhibition of JNK did not, indicating that positive feedback regulation between ERK activation and ROS generation triggers ER stress in response to combined treatment. Furthermore, prevention of ER stress completely blocked combination treatment-induced JNK activation and subsequent apoptotic cell death. In addition, combined treatment efficiently induced the mitochondrial translocation of cleaved Bax, disrupted mitochondrial membrane potential, and the nuclear translocation of AIF, all of which were efficiently blocked by a JNK inhibitor. Caspases 3, 8 and 9 were activated by combined treatment but inhibition of these caspases did not abolish apoptosis indicating caspase activation played a minor role in the induction of apoptosis.

**Conclusions/Significance:**

β-lap causes NQO1-dependent radiosensitization of cancer cells. When NQO1^+^ cells are treated with combination of IR and β-lap, positive feedback regulation between ERK and ROS leads to ER stress causing JNK activation and mitochondrial translocation of cleaved Bax. The resultant decrease in mitochondrial membrane leads to translocation of AIF and apoptosis.

## Introduction

β-lapachone (β-lap) is a bioreductive agent that has been shown to possess strong anti-cancer activity both *in vitro* and *in vivo* [1]–[3]. The anti-cancer activity of β-lap has been shown to be due to the two-electron reduction of β-lap mediated by NAD(P)H:quinone oxidoreductase (NQO1, DT-diaphorase) using NADH or NAD(P)H as electron sources [1]–[3]. Because NQO1 is expressed more abundantly in a variety of human solid cancers than in normal tissues [1]–[3], β-lap can selectively kill human cancer cells. β-lap has also been shown to sensitize cancer cells to ionizing radiation (IR) [4]. However, the precise underlying this radiosensitizing mechanism has not yet been elucidated.

Futile cycling between the oxidized and reduced forms of β-lap has been shown to cause progressive depletion of NADH and NAD(P)H, which, in turn induces massive release of Ca^2+^ from the endoplasmic reticulum (ER) into the cytosol, leading to activation of the Ca^2+^-dependent proteinase, calpain and subsequent apoptotic cell death [1], [2], [5]. Furthermore, redox cycling caused by one-electron reduced β-lap (i.e., the semiquinone form of β-lap), the intermediate between two-electron β-lap and the oxidized form of β-lap can trigger the activation of cell death pathways [1]. Recent studies suggest that generation of reactive oxygen species (ROS) by diverse cell death stimuli does not only initiate cascades of cell death signals but also directly lead to DNA damage [6]–[10]. However, the signaling pathways activated by ROS in cells treated with β-lap have not yet been clearly delineated.

Although β-lap was demonstrated to activate mitogen-activated protein kinases (MAPKs) in cancer cells, and thereby induce apoptotic death [11], the signaling pathways involved in the activation of MAPKs caused by β-lap, and the precise role of MAPK activation in β-lap-induced apoptosis have not been clarified.

The mitochondrial cell death pathway is regulated by the ratio of pro- to anti-apoptotic proteins, including members of the Bcl-2 family. Among these family members, Bax or Bak plays a key role in the loss of mitochondrial transmembrane potential [12]. Upon delivery of an apoptotic stimulus, cytosolic Bax translocates to the outer mitochondrial membrane, where it oligomerizes to form homodimers, creating pores that expedite the release of cytochrome *c*, apoptosis-inducing factor (AIF), and endonuclease G (Endo G) from the intermembrane space of the mitochondrion into the cytosol [7].

In this study, we investigated the mechanism by which β-lap modulates the response of breast cancer cells to radiation. We demonstrate here that combined treatment of IR and β-lap synergistically increases clonogenic and apoptotic cell death in an NQO1-dependent manner. We also show that the generation of ROS, induction of ER stress, and activation of extracellular-regulated kinase (ERK) and c-Jun N-terminal kinase (JNK) by combined treatment are crucial for mitochondrial apoptotic cell death. Specifically, we concluded that formation of a positive feedback loop between ERK activation and ROS generation is needed for combined treatment-induced ER stress, which leads to JNK activation and subsequent mitochondrial apoptotic cell death. Our data provide a potential mechanism to account for the radiosensitizing effect of β-lap, and insight gained in the present study will lead to advances in the clinical application of combined treatment with IR and β-lap in therapies based on NQO1-directed tumor targeting.

## Results

### Combined treatment with IR and β-lap increases clonogenic and apoptotic cell death in an NQO1-dependent manner

**Figure 1:**
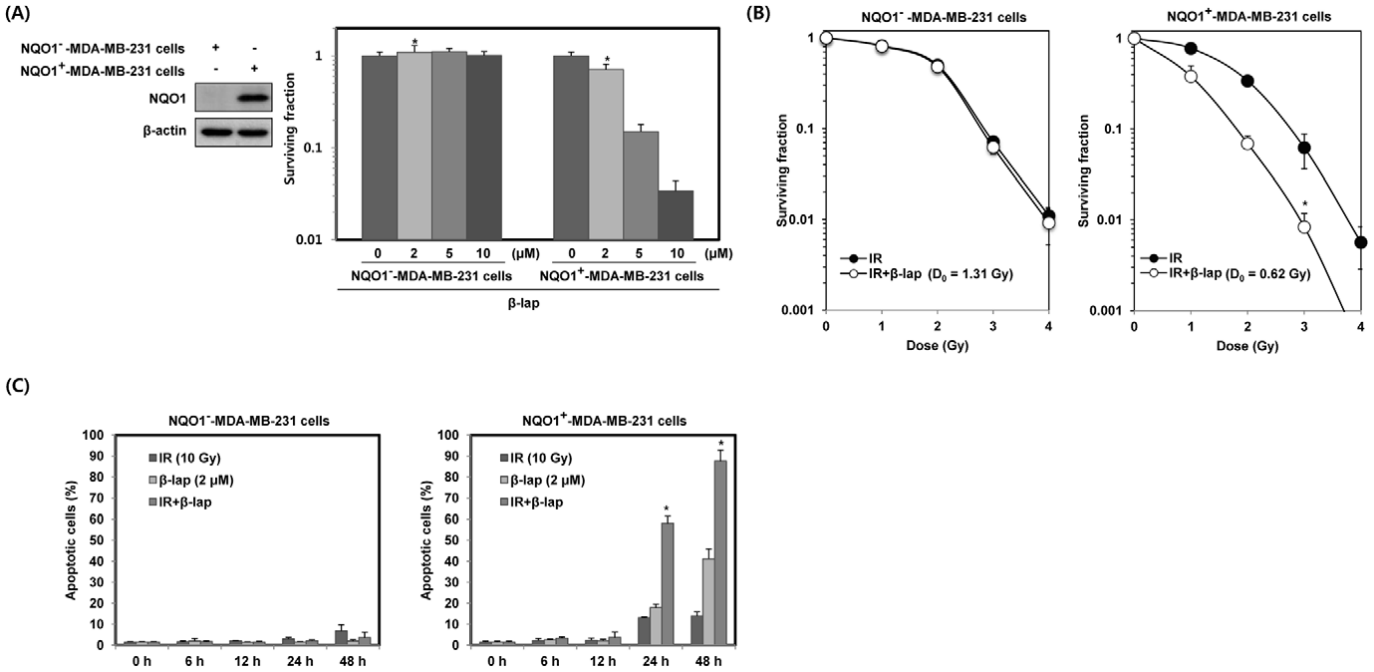
β-lap induces radiosensitization in an NQO1-dependent manner. (A) NQO1^−^ or NQO1^+^-MDA-MB-231 cells were treated with various concentrations of β-lap. Cells were allowed to grow for 10 to 14 days and were stained with 0.5% crystal violet and scored for colony formation. Results from three independent experiments are expressed as means ± SEM. *Significant difference between NQO1^−^- and NQO1^+^-MDA-MB-231 cells after β-lap treatment at p<0.05. (B) Cells were treated with 2 µM β-lap and then exposed to increasing doses of IR 30 min after treatment with β-lap. After 14 days, cells were scored for colony formation. Results from three independent experiments are expressed as means ± SEMs. (C) Cells were treated with IR alone, β-lap alone or the combination of IR and β-lap for the indicated times. The percentage of cells with sub-G1 DNA content was determined by flow cytometry. Results from three independent experiments are expressed as means ± SEMs.

To determine the effect of β-lap on clonogenic cell survival, we treated parental NQO1^−^-MDA-MB-231 cells deficient in NQO1, or NQO1^+^-MDA-MB-231 cells possessing abundant expression of NQO1, with different doses of β-lap. As shown in Figure 1A, a dose dependent increase in clonogenic cell death following β-lap treatment was obvious in NQO1^+^-MDA-MB-231 cells, but not in parental NQO1^−^-MDA-MB-231 cells, indicating NQO1 plays a crucial role for the β-lap-induced cell death.

We analyzed the effect of β-lap on the IR-induced clonogenic death of NQO1^−^-MDA-MB-231 and NQO1^+^-MDA-MB-231 cells. Cells were exposed to different doses of IR in the presence or absence of 2 µM β-lap and the clonogenic survival was determined as shown in Fig. 1B. The radiation survival curves for combined treatment were normalized for the death by β-lap alone. The radiation survival curve for combined treatment with IR and β-lap was identical in NQO1^+^-MDA-MB-231 cells. On the other hand, the radiation survival curve for combined treatment of NQO1^+^-MDA-MB-231 cells was significantly steeper particularly at the lower radiation doses than that for IR treatment alone resulting in significant decrease in the shoulder of the survival curve. The reduction of shoulder indicated that β-lap inhibited the repair of sublethal radiation damage in NQO1^+^ cells but not in NQO1^−^ cells.

We investigated the combined effect of IR and β-lap on apoptotic cell death in NQO1^−^-MDA-MB-231 and NQO1^+^-MDA-MB-231 cells. Cells were treated with 10 Gy of IR alone, 2 µM β-lap alone, or combination of IR and β-lap for various lengths of time, and apoptotic cell death was assessed. While apoptosis of NQO1^+^-MDA-MB-231 cells induced by IR alone was about 13 and 14% at 24 and 48 h, respectively, β-lap alone-induced apoptosis in about 18 and 40% of cells at 24 and 48 h, respectively (Fig. 1C). Combined treatment with IR and β-lap remarkably increased apoptotic cell death in NQO1^+^-MDA-MB-231 cells; about 58 and 87% of cells were apoptotic at 24 and 48 h, respectively. On the contrary, in NQO1^−^-MDA-MB-231 cells, no significant apoptosis occurred after treating with IR or β-lap alone or even with combination of IR and β-lap, most likely due to NQO1 deficiency and a mutant form of p53 expressed in NQO1^−^-MDA-MB-231 cells [13].

### Combined treatment with IR and β-lap markedly induces ROS generation in NQO1^+^-MDA-MB-231 cells

**Figure 2:**
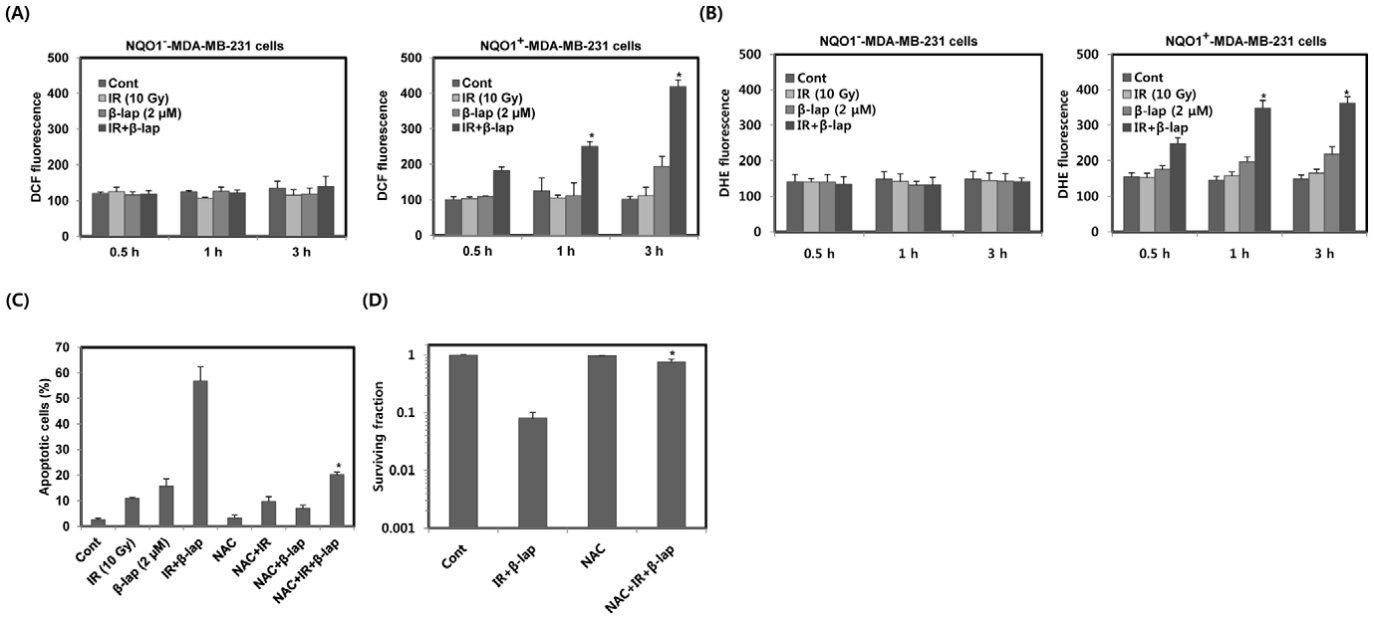
β-lap in combination with IR enhances ROS generation and leads to apoptotic cell death. (A) and (B) Cells were treated with IR alone, β-lap alone or combination of IR and β-lap for the indicated times. After 3 h, the cells were incubated with 10 µM H2DCF-DA and 4 µM DHE, respectively, for 30 min and then analyzed by flow cytometry. Results from three independent experiments are expressed as means ± SEMs. (C) NQO1^+^-MDA-MB-231 cells were treated with IR alone, β-lap alone or combination of IR and β-lap for 24 h in the presence or absence of NAC (10 mM). After 24 h, the percentage of cells with sub-G1 DNA content was determined by flow cytometry. Results from three independent experiments are expressed as means ± SEMs. (D) NQO1^+^-MDA-MB-231 cells were treated with combination of IR (2 Gy) and β-lap (2 µM) in the presence or absence of NAC (10 mM). Cells were allowed to grow for 10 to 14 days and were stained with 0.5% crystal violet and scored for colony formation. Results from three independent experiments are expressed as means ± SEM. *Significant difference between cells in the presence or absence of NAC after combined treatment with IR and β-lap, at p<0.05.

We investigated the involvement of ROS in combined treatment-induced apoptotic cell death in NQO1^−^-MDA-MB-231 and NQO1^+^-MDA-MB-231 cells. We first measured ROS with DCF staining. Compared to individual treatment with IR or β-lap, combined treatment caused far more increase in ROS levels for 3 h in NQO1^+^-MDA-MB-231 cells but not in NQO1^−^-MDA-MB-231 (Fig. 2A). We further confirmed the ROS generation by combined treatment with IR and β-lap using another ROS detection dye, dihydroethidium (DHE). As shown in Figure 2B, we observed marked increase in ROS generation in NQO1^+^-MDA-MB-231 cells but not in NQO1^−^-MDA-MB-231 cells after combined treatment with IR and β-lap. To determine whether the increase in intracellular ROS levels was directly related to apoptotic cell death, we pretreated NQO1^+^-MDA-MB-231 cells with the antioxidant NAC before exposing cells to IR and β-lap. As shown in Figure 2C, pretreatment with NAC significantly reduced the apoptotic cell death caused by combined treatment with IR and β-lap. Furthermore, pretreatment with NAC effectively attenuated the clonogenic cell death caused by combined treatment with IR and β-lap (Fig. 2D). These observations suggest that induction of ROS critically contributes to the apoptotic and clonogenic cell death caused by combined treatment with IR and β-lap in NQO1^+^-MDA-MB-231 cells.

### β-lap in combination with IR rapidly activates ERK and JNK, leading to apoptotic cell death

**Figure 3:**
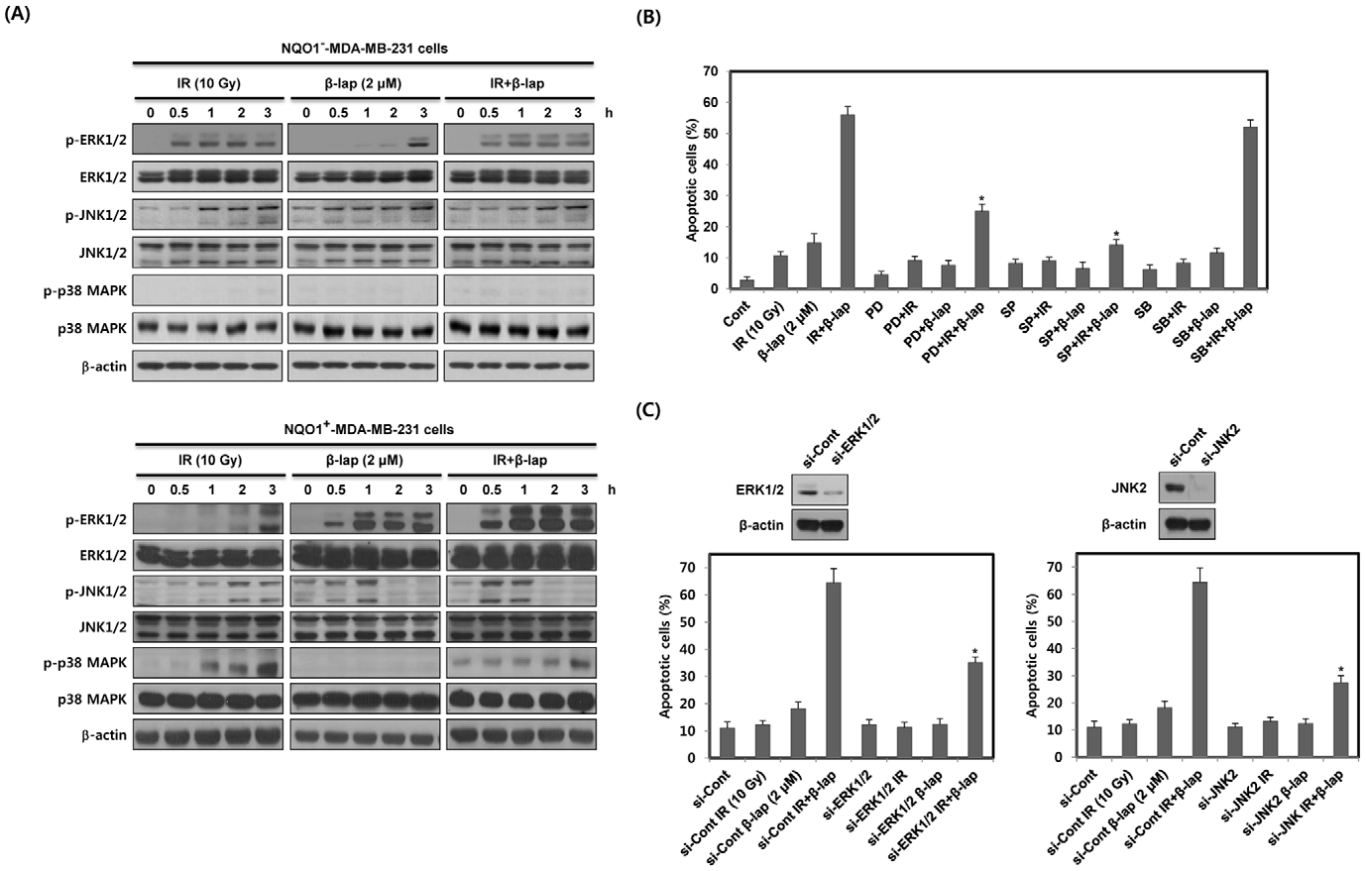
β-lap in combination with IR rapidly activates ERK and JNK. (A) NQO1^−^ or NQO1^+^-MDA-MB-231 cells were treated with IR alone, β-lap alone or combination of IR and β-lap for the indicated times. The data represent a typical experiment conducted three times with similar results. (B) and (C) NQO1^+^-MDA-MB-231 cells were treated with IR alone, β-lap alone or combination of IR and β-lap for 24 h in the presence or absence of PD98059 (30 µM), SP600125 (30 µM), SB203580 (30 µM), or siRNA targeting ERK1/2 or JNK2. The percentage of cells with sub-G1 DNA content was determined by flow cytometry. Results from three independent experiments are expressed as means ± SEMs.

To reveal the potential involvement of MAPKs in the apoptotic cell death caused by combined treatment with IR and β-lap, we studied the levels of the activated forms of MAPKs in NQO1^−^-MDA-MB-231 and NQO1^+^-MDA-MB-231 cells by Western blot analysis using anti-phospho antibodies. As shown in Figure 3A, in NQO1^−^-MDA-MB-231 cells, IR alone slightly increased phosphorylation of ERK and JNK while β-lap alone caused little change in the levels of phosphorylated ERK and JNK. The effect of combined treatment was similar to that of IR alone. The phosphorylation of p38 MAPK in NQO1^−^-MDA-MB-231 cells was negative after either single or combined treatments with IR and β-lap. However, in NQO^+^-MDA-MB-231 cells, combined treatment led to a rapid up-regulation of phosphorylated ERK and JNK as compared with the treatment with IR alone or β-lap alone (Fig. 3A). ERK activation was apparent at 0.5 h, and remained elevated for 3 h after combined treatment. In addition, JNK activation started to increase within 0.5 h but diminished 2 h after combined treatment in NQO1^+^-MDA-MB-231 cells. The total cellular levels of ERK and JNK remained constant. IR treatment increased phosphorylation of p38 MAPK, while β-lap treatment caused no change in phosphorylation of p38 MAPK. The phosphorylation level of p38 MAPK after combined treatment was lower than that after treatment with IR alone, indicating β-lap suppressed IR-induced activation of p38 MAPK in NQO1^+^-MDA-MB-231 cells (Fig. 3A). To examine the relationship between the activation of MAPKs and apoptotic cell death, we pretreated NQO1^+^-MDA-MB-231 cells with SP600125, PD98059, or SB203580, inhibitors of JNK, MEK/ERK, and p38 MAPK, respectively, prior to treatment with IR and β-lap. As shown in Figure 3B, PD98059 and SP600125 effectively reduced the apoptotic cell death caused by combined treatment from 56% to 24% and 13%, respectively, whereas SB203580 was ineffective. To further investigate the involvement of ERK and JNK2 in the apoptotic cell death caused by combined treatment with IR and β-lap, we pretreated NQO1^+^-MDA-MB-231 cells with siRNAs targeting ERK and JNK2. Consistent with the results obtained with pharmacological inhibitors, specific inhibition of ERK and JNK2 with siRNAs almost completely abolished the apoptotic cell death caused by combined treatment (Fig. 3C). These results demonstrate that ERK and JNK act as important mediators of apoptotic cell death induced by combined treatment with IR and β-lap in NQO1^+^-MDA-MB-231 cells.

### Positive feedback regulation between ROS and ERK induced by combined treatment with IR and β-lap is essential for the activation of JNK

**Figure 4:**
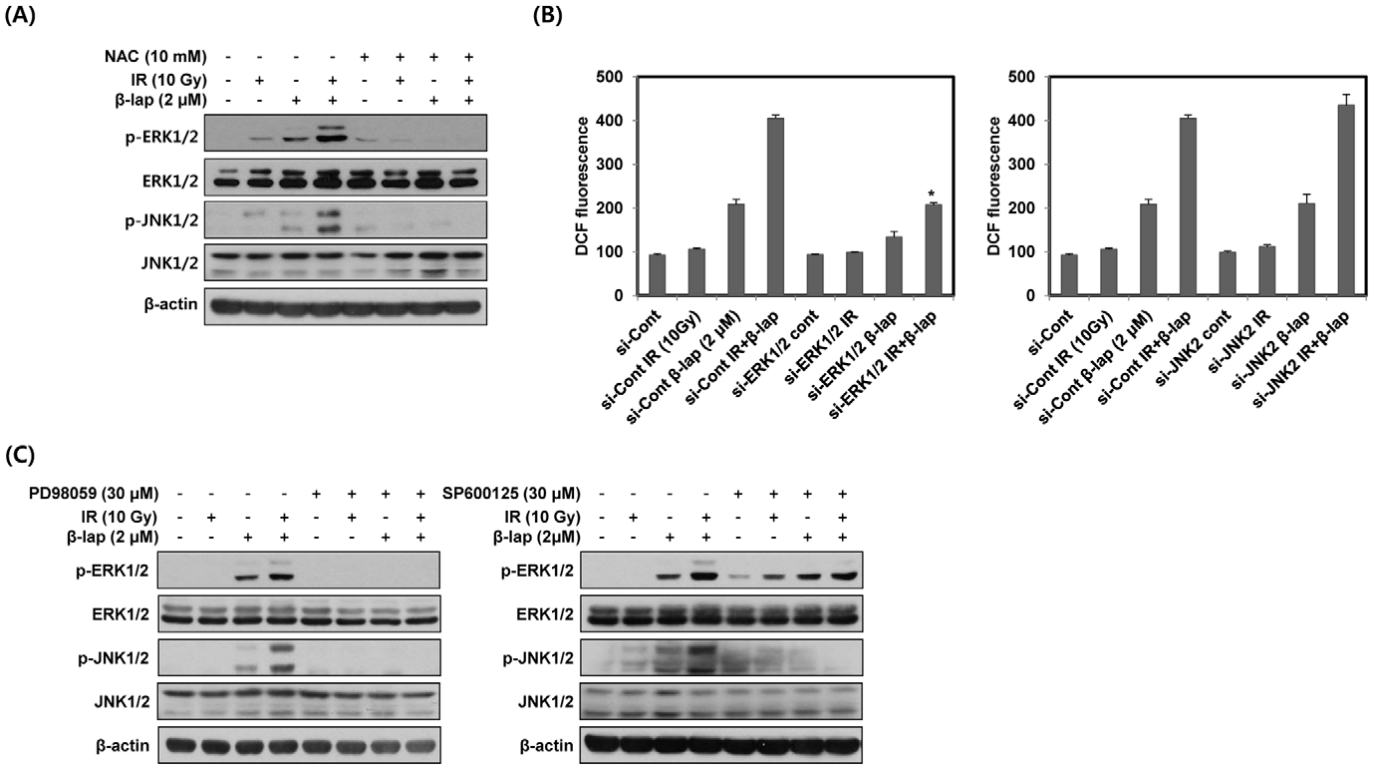
β-lap in combination with IR induces positive feedback regulation between ERK and ROS. (A) NQO1^+^-MDA-MB-231 cells were treated with IR alone, β-lap alone or combination of IR and β-lap for 30 min in the presence or absence of NAC. The data represent a typical experiment conducted three times with similar results. (B) NQO1^+^-MDA-MB-231 cells were treated with IR alone, β-lap alone or combination of IR and β-lap for 3 h in the presence or absence of siRNA targeting ERK1/2 or JNK2. After 3 h, the cells were incubated with 10 µM H2DCF-DA for 30 min and then analyzed by flow cytometry. Results from three independent experiments are expressed as means ± SEMs. (C) NQO1^+^-MDA-MB-231 cells were treated with IR alone, β-lap alone or combination of IR and β-lap for 30 min in the presence or absence of PD98059 or SP600125. The data represent a typical experiment conducted three times with similar results.

To determine whether ROS generation is involved in the activation of ERK and JNK by combined treatment with IR and β-lap, we pretreated NQO1^+^-MDA-MB-231 cells with the antioxidant NAC prior to treatment with IR and β-lap. As shown in Figure 4A, the activation of ERK and JNK by combined treatment was completely blocked by pretreatment with NAC. We next examined the contribution of ERK and JNK to ROS generation caused by combined treatment. We treated NQO1^+^-MDA-MB-231 cells with siRNAs targeting ERK and JNK2 prior to treatment with IR and β-lap. As shown in Figure 4B, siRNA-mediated ERK knockdown effectively blocked ROS generation, as determined with DCF, induced by β-lap alone and to an even greater extent by combined treatment, whereas siRNA targeting JNK2 did not attenuate ROS generation.

To further elucidate the possibility of cross-talk between ERK and JNK, we pretreated NQO1^+^-MDA-MB-231 cells with PD98059 or SP600125. As shown in Figure 4C, activation of ERK and JNK by combined treatment was completely abrogated by pretreatment with PD98059 and SP600125, respectively. Moreover, PD98059 effectively blocked combination treatment-induced activation of JNK, whereas SP600125 did not attenuate the activation of ERK, indicating that ERK is an upstream activator of JNK (Fig. 4C). These results indicated that β-lap in combination with IR leads to positive feedback regulation between ROS and ERK activation that may contribute to JNK activation.

### ROS generation induced by combined treatment with IR and β-lap is required for the induction of ER stress

**Figure 5:**
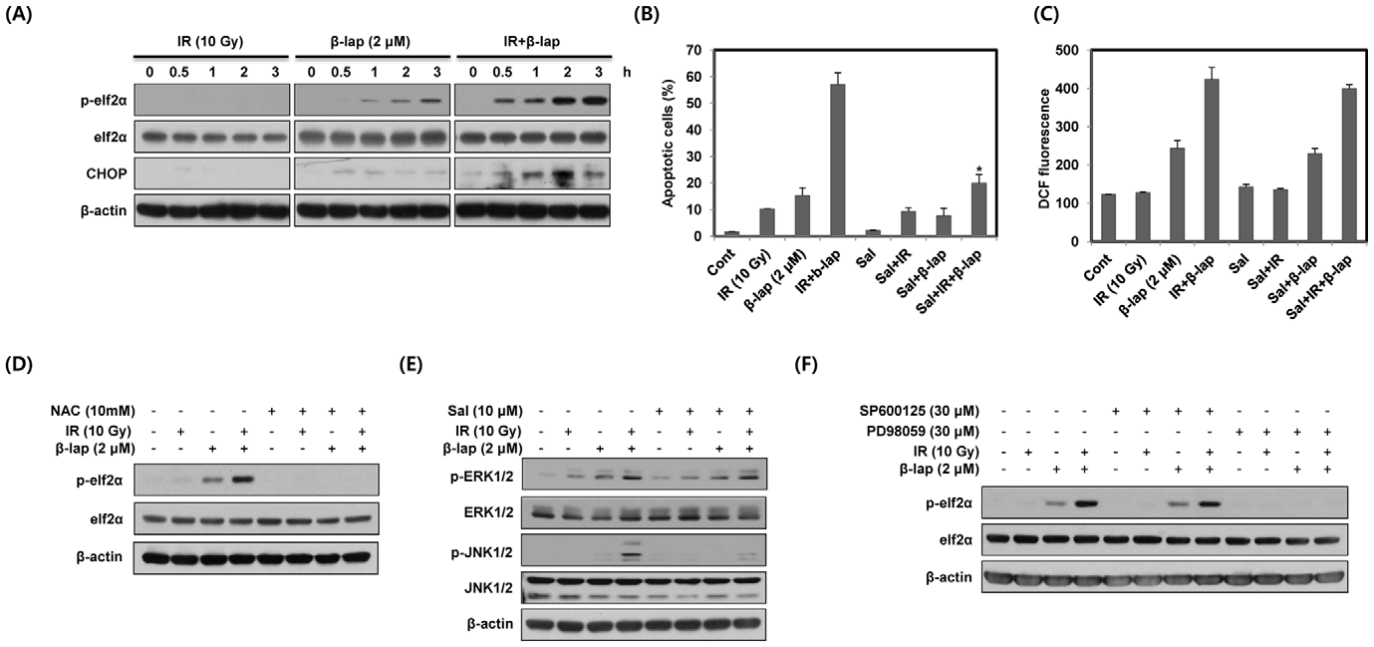
The induction of ER stress by combined treatment with IR and β-lap is required for JNK activation. (A) NQO1^+^-MDA-MB-231 cells were treated with IR alone, β-lap alone or combination of IR and β-lap for the indicated times. Cell lysates were subjected to Western blot analysis. The data represent a typical experiment conducted three times with similar results. (B) NQO1^+^-MDA-MB-231 cells were treated with IR alone, β-lap alone or combination of IR and β-lap for 24 h in the presence or absence of Sal (10 µM). After 24 h, the percentage of cells with sub-G1 DNA content was determined by flow cytometry. Results from three independent experiments are expressed as means ± SEMs. (C) NQO1^+^- MDA-MB-231 cells were treated with IR alone β-lap alone or combination of IR and β-lap for 3 h in the presence or absence of Sal (10 µM). After 3 h, the cells were incubated with 10 µM H2DCF-DA for 30 min and then analyzed by flow cytometry. Results from three independent experiments are expressed as means ± SEMs. (D) NQO1^+^-MDA-MB-231 cells were treated with IR alone, β-lap alone or combination of IR and β-lap for 30 min in the presence or absence of NAC. The data represent a typical experiment conducted three times with similar results. (E) and (F) NQO1^+^-MDA-MB-231 cells were treated with IR alone, β-lap alone or combination of IR and β-lap for 30 min in the presence or absence of Sal, SP600125 or PD98059. The data represent a typical experiment conducted three times with similar results.

We next investigated whether combined treatment with IR and β-lap induces ER stress in NQO1^+^-MDA-MB-231 cells using phosphorylation of eIF2α and CHOP expression. As shown in Figure 5A, IR alone did not alter phosphorylated eIF2α (p-eIF2α) levels and CHOP expression, and β-lap alone slightly increased p-eIF2α and CHOP expression. However, combined treatment markedly induced p-eIF2α and increased CHOP expression level (Fig. 5A). To further examine the involvement of ER stress in apoptotic cell death induced by combined treatment with IR and β-lap, we pretreated NQO1^+^-MDA-MB-231 cells with Salubrinal (Sal), an ER stress inhibitor. As shown in Figure 5B, Sal effectively suppressed combination treatment-induced apoptotic cell death. These results indicate that ER stress is a major contributor to combined treatment-induced apoptotic cell death in NQO1^+^-MDA-MB-231 cells.

We next investigated the relationship between ROS generation and ER stress in NQO1^+^-MDA-MB-231 cells treated with combination of IR and β-lap. As shown in Figure 5C, pretreatment with Sal did not attenuate the ROS generation caused by combined treatment, whereas pretreatment with NAC effectively prevented the phosphorylation of eIF2α (Fig. 5D), indicating that ROS generation induced by combined treatment is required for the induction of ER stress.

### ER stress induced by combined treatment with IR and β-lap is required for the activation of JNK

To analyze the relationship between ER stress and MAPKs (ERK and JNK), we first examined the effect of Sal on the activation of ERK and JNK. As shown in Figure 5E, pretreatment with Sal efficiently suppressed the activation of JNK induced by combined treatment, but did not attenuate the activation of ERK. To further establish the critical role of MAPKs in the induction of ER stress, we pretreated NQO1^+^-MDA-MB-231 cells with PD98059 or SP600125. As shown in Figure 5F, combined treatment-induced phosphorylation of eIF2α was completely blocked by PD98059, but not by SP600125. These results indicate that ERK is an upstream activator of ER stress responsible for JNK activation in response to combined treatment with IR and β-lap.

### Combined treatment with IR and β-lap induces apoptotic cell death through the nuclear translocation of AIF

**Figure 6:**
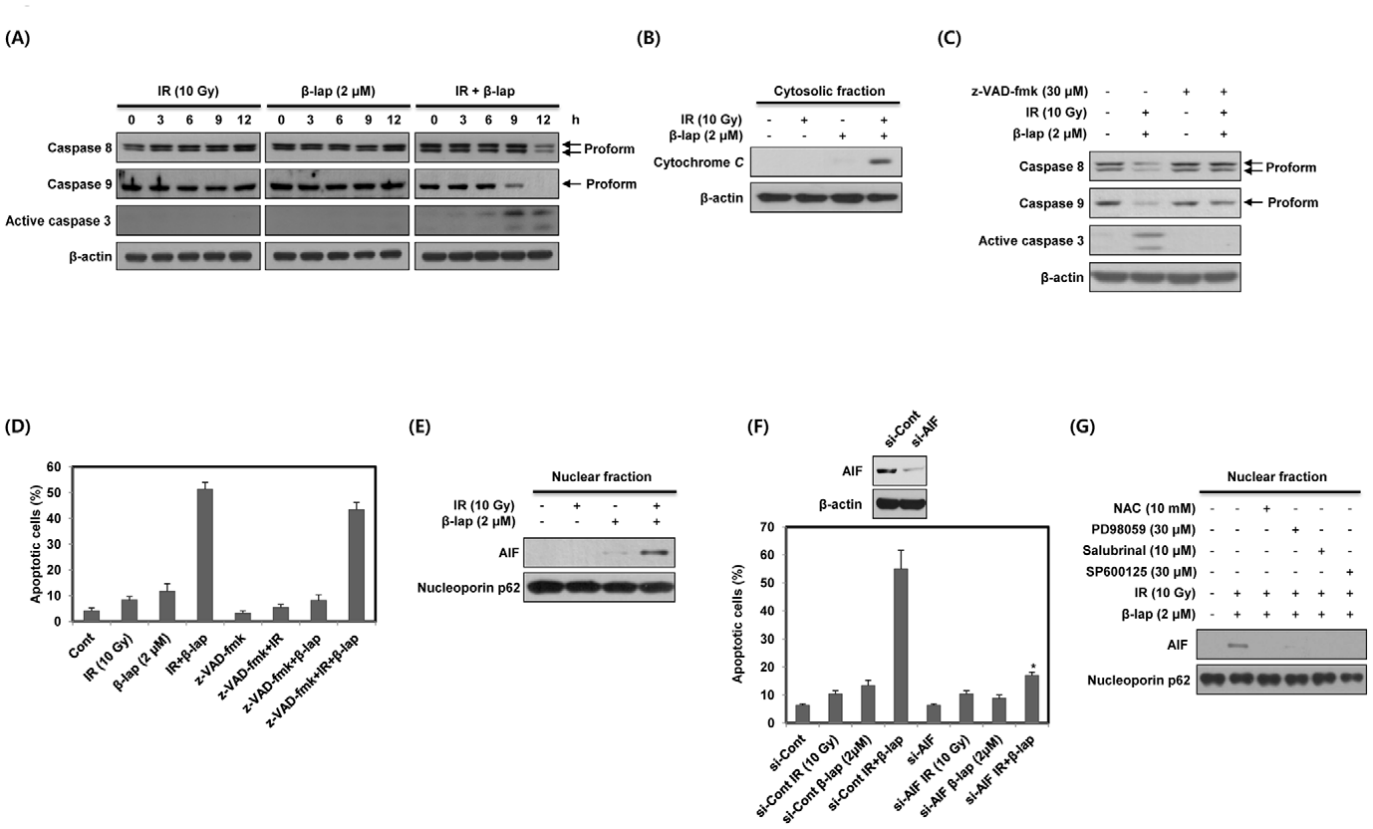
β-lap in combination with IR induces apoptotic cell death via nuclear translocation of AIF. (A) NQO1^+^-MDA-MB-231 cells were treated with IR alone, β-lap alone or combination of IR and β-lap for the indicated times. The data represent a typical experiment conducted three times with similar results. (B) NQO1^+^-MDA-MB-231 cells were treated with IR alone, β-lap alone or combination of IR and β-lap for 12 h. Cytosolic fractions from NQO1^+^-MDA-MB-231 cells were prepared and subjected to Western blot analysis. The data are representative a typical experiment conducted three times. The data represent a typical experiment conducted three times with similar results. (C) NQO1^+^-MDA-MB-231 cells were treated with combination of IR and β-lap for 12 h in the presence or absence of z-VAD-fmk. The data represent a typical experiment conducted three times with similar results. (D) NQO1^+^-MDA-MB-231 cells were treated with IR alone, β-lap alone or combination of IR and β-lap for 24 h in the presence or absence of z-VAD-fmk (30 µM). After 24 h, the percentage of the cells with sub-G1 DNA content was determined by flow cytometry. Results from three independent experiments are expressed as means ± SEMs. (E) NQO1^+^-MDA-MB-231 cells were treated with IR alone, β-lap alone or combination of IR and β-lap for 24 h. Nuclear fractions from NQO1^+^-MDA-MB-231 cells were prepared and subjected to Western blot analysis. The data are representative a typical experiment conducted three times. (F) NQO1^+^-MDA-MB-231 cells were treated with IR alone, β-lap alone or combination of IR and β-lap for 24 h in the presence or absence of siRNA targeting AIF. After 24 h, the percentage of the cells with sub-G1 DNA content was determined by flow cytometry. Results from three independent experiments are expressed as means ± SEMs. (G) NQO1^+^-MDA-MB-231 cells were treated with combination of IR and β-lap for 24 h in the presence or absence of NAC, PD98059, Sal or SP600125. Nuclear fractions were prepared and subjected to Western blot analysis. The data are representative a typical experiment conducted three times.

Because activation of the caspase pathway is an important mechanism for inducing apoptotic cell death [14], we investigated the involvement of caspases in the apoptotic response to combined treatment of NQO1^+^-MDA-MB-231 cells with IR and β-lap. As shown in Figure 6A, combined treatment led to activations of caspase-8, -9 and -3. Note that activation of caspase 9 and 3 occurred prior to activation of caspase 8. IR or β-lap alone caused no evident activations of these caspases. Cytochrome c released from the mitochondria has been reported to form a complex with procaspase-9 and apoptotic protease-activating factor-1 (Apaf-1), resulting in activation of procaspase-9 [14]. Therefore, we determined whether combined treatment with IR and β-lap induces the release of cytochrome c from the mitochondria to the cytosol. As shown in Figure 6B, combined treatment efficiently raised the level of cytochrome c within the cytosolic fraction for 12 h, whereas IR or β-lap alone did not. We then studied the requirement of caspase for combined treatment-induced apoptosis using a broad-spectrum caspase inhibitor, z-VAD-fmk. Figure 6C shows that z-VAD-fmk effectively prevented activation of caspases caused by combined treatment. Surprisingly, however, z-VAD-fmk only partially reduced combined treatment-induced apoptotic cell death from about 51% to 42% (Fig. 6D). These results indicate that the apoptotic cell death caused by combined treatment with IR and β-lap occurs, even though activation of caspases is inhibited.

Because AIF, a mitochondria-localized flavoprotein, is known to be involved in the induction of caspase-independent apoptotic cell death [7], we next investigated whether AIF plays a role in the induction of cell death by combined treatment with IR and β-lap. AIF is released from the mitochondria in response to cell death stimuli, subsequently translocates to the nucleus, where it causes DNA fragmentation [7]. Subcellular fractionation showed that combined treatment dramatically induced the nuclear translocation of AIF, while IR or β-lap alone caused little increase in AIF in the nucleus (Fig. 6E). Moreover, siRNA-mediated AIF knockdown effectively reduced combination treatment-induced apoptotic cell death from 55% to almost control levels (Fig. 6F). These results suggest that nuclear translocation of AIF is required for the induction of apoptotic cell death by combined treatment with IR and β-lap.

To further define the roles of ROS, ERK, ER stress and JNK in nuclear translocation of AIF after combined treatment with IR and β-lap, we pretreated NQO1^+^-MDA-MB-231 cells with the corresponding inhibitors, NAC, PD98059, Sal or SP600125, and examine the subcellular localization of AIF. As shown in Figure 6G, the nuclear translocation of AIF induced by combined treatment was completely blocked by pretreatment with NAC, PD98059, Sal or SP600125. These results show that ROS, ERK, ER stress and JNK are upstream activators of AIF-mediated cell death induced by combined treatment with IR and β-lap.

### Combined treatment with IR and β-lap induces mitochondrial translocation of cleaved Bax and disruption of mitochondrial transmembrane potential

**Figure 7:**
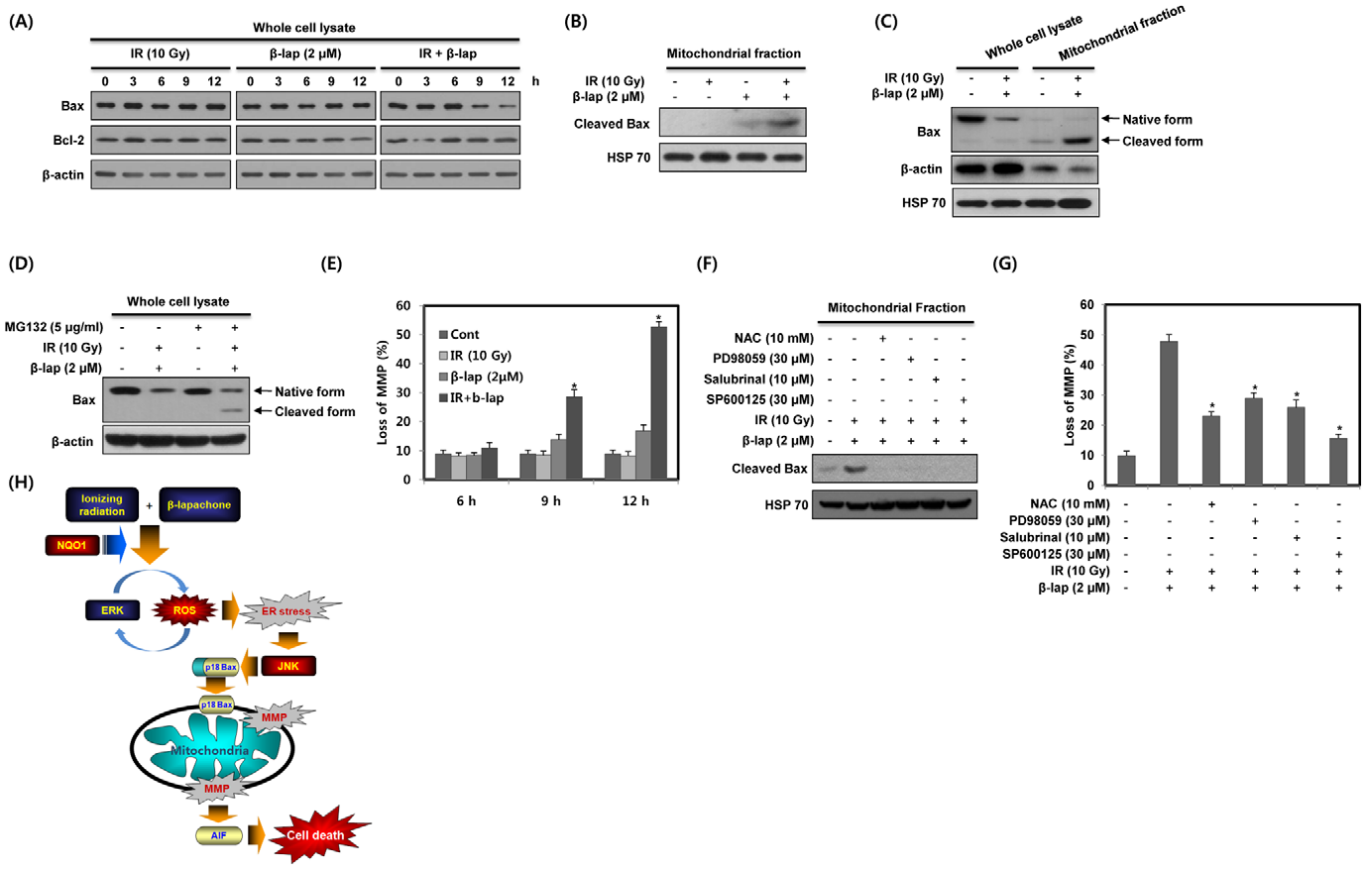
β-lap in combination with IR causes Bax cleavage and translocation of cleaved Bax to mitochondria. (A) NQO1^+^-MDA-MB-231 cells were treated with IR alone, β-lap alone or combination of IR and β-lap for the indicated times. Cell lysates were subjected to Western blot analysis. The data represent a typical experiment conducted three times with similar results. (B) NQO1^+^-MDA-MB-231 cells were treated with IR alone, β-lap alone or combination of IR and β-lap for 12 h. Mitochondrial fractions of NQO1^+^-MDA-MB-231 cells were prepared and subjected to Western blot analysis. The data are representative a typical experiment conducted three times. (C) NQO1^+^-MDA-MB-231 cells were treated with IR alone, β-lap alone or combination of IR and β-lap for 12 h. Whole cell lysates and mitochondrial fractions of NQO1^+^-MDA-MB-231 cells were prepared and subjected to Western blot analysis. The data are representative a typical experiment conducted three times. (D) NQO1^+^-MDA-MB-231 cells were treated with combination of IR and β-lap for 24 h in the presence or absence of MG132. The data are representative a typical experiment conducted three times. (E) NQO1^+^-MDA-MB-231 cells were treated with IR alone, β-lap alone or combination of IR and β-lap for the indicated times. After 12 h, the concentration of retained DiOC_6_(3) in cells was measured by flow cytometry. Results from three independent experiments are expressed as means ± SEMs. (F) NQO1^+^-MDA-MB-231 cells were treated with combination of IR and β-lap for 12 h in the presence or absence of NAC, PD98059, Sal or SP600125. Mitochondrial fractions were prepared and subjected to Western blot analysis. The data are representative a typical experiment conducted three times. (G) NQO1^+^-MDA-MB-231 cells were treated with combination of IR and β-lap for 12 h in the presence or absence of NAC, PD98059, Sal or SP600125. After 12 h, the concentration of retained DiOC_6_(3) in cells was measured by flow cytometry. Results from three independent experiments are expressed as means ± SEMs. (H) Schematic model of combined treatment (IR+β-lap)-induced apoptotic cell death. Combined treatment with IR and β-lap increases mitochondrial apoptotic cell death in an NQO1 dependent manner. As described in detail in the text, positive feedback regulation between ERK and ROS induced by combined treatment plays a critical role in the induction of ER stress. This enhanced ER stress is required for JNK activation, which leads to subsequent mitochondrial apoptotic cell death. Moreover, JNK activation induces cleavage of Bax and mitochondrial translocation of cleaved Bax, which causes loss of mitochondrial transmembrane potential and consequent release of AIF.

To determine the role of mitochondrial pathway in combined treatment-induced apoptosis in NQO1^+^-MDA-MB-231 cells, we first examined changes in Bcl-2 and Bax expression levels, and mitochondrial transmembrane potential. As shown in Figure 7A, Bax levels were markedly reduced in response to combined treatment with IR and β-lap, but not in response to individual treatment with IR or β-lap. The expression levels of Bcl-2 were unchanged by individual or combined treatment. It has been reported that Bax is cleaved from a 21 kDa native form to an 18 kDa fragment in response to death stimuli [15], [16]. Because translocation of 18 kDa cleaved Bax from the cytosol to mitochondria has been reported to induce a decline in mitochondrial transmembrane potential and subsequent release of proapoptogenic proteins from mitochondria [15], [16], we investigated whether combined treatment induces mitochondrial translocation of cleaved Bax. As shown in Figure 7B and C, combined treatment with IR and β-lap more effectively increased the levels of 18 kDa cleaved Bax within the mitochondrial fraction than individual treatment with IR or β-lap. However, 18 kDa form of Bax was undetectable in whole cell lysates after combined treatment (Fig. 7C). Because a cathepsin-like protease has been suggested to be involved in the rapid degradation of 18 kDa form of Bax in the cytosol [17], we pretreated cells with the protease inhibitor MG132 to know why cleaved Bax was absent in whole cell lysate after combined treatment with IR and β-lap. As shown in Figure 7D, when cells were treated with combination of IR and β-lap in the presence of MG132, cleaved Bax was detected in whole cell lysate, indicating that cleaved Bax is readily degraded in the cytosol after combined treatment with IR and β-lap. Thus, the 18 kDa form of Bax may be unstable in the cytosol but stable in the mitochondrial fraction after combined treatment with IR and β-lap. Furthermore, as shown in Figure 7E, combined treatment significantly disrupted mitochondrial transmembrane potential in a time-dependent manner, coinciding with the observed changes in Bax levels. Taken together, these results demonstrate that combined treatment-induced cell death involves an alteration in mitochondrial transmembrane potential mediated by intracellular redistribution of cleaved Bax.

We next examined the role of ROS, ERK, ER stress and JNK in the mitochondrial translocation of cleaved Bax and disruption of mitochondrial transmembrane potential induced by combined treatment with IR and β-lap. Pretreatment with NAC, PD98059, Sal or SP600125 significantly attenuated combination treatment-induced mitochondrial translocation of cleaved Bax and disruption of mitochondrial transmembrane potential (Fig. 7F and G). These results indicate that ROS, ERK, ER stress and JNK are upstream activators of the mitochondrial apoptotic cell death induced by combined treatment with IR and β-lap.

## Discussion

The aim of our study was to elucidate signaling pathways involved in β-lap-induced radiosensitization in human breast cancer cells. We found that β-lap in combination with IR caused cell death significantly greater than that by individual treatment alone or more than additive in an NQO1-dependent manner, and showed that the induction of ER stress by the positive feedback regulation between ERK and ROS caused JNK activation, thereby contributing to combined treatment-induced mitochondrial apoptotic cell death.

The mechanism of the anti-cancer effect of β-lap alone or in combination with other treatment modalities has been reported in recent years [3], [4], [18]–[22]. Interestingly, β-lap has been found to potentiate the effect of taxol, mitomycin C, genistein, cisplatin, and IR on human cancer cells [3], [4], [18]–[22]. We have previously reported that IR sensitized cells to β-lap by up-regulating NQO1 expression and acitivity [3], [18]. In the present study, we observed that β-lap in combination with IR increases clonogenic and apoptotic cell death of MDA-MB-231 human breast cancer cells in an NOQ1-dependent manner (Fig. 1). It has been demonstrated that β-lap induces radiosenstization by inhibiting the repair of DNA damage caused by IR [4]. Recently, hyperactivation of poly (ADP-ribose) polymerase-1 (PARP-1) has been suggested to play an important role in β-lap-induced radiosensitization in prostate cancer cells that overexpress NQO1 [23]. Furthermore, previously, it has been reported that ionizing radiation causes a long lasting elevation of NQO1 activity, which can participate in stabilization of p53 protein by interfering with 20S proteasome-mediated p53 degradation [24], [25]. The stabilized p53 not only activate the transcriptional expression of various genes involved in apoptosis and cell cycle arrest by playing as a transcription factor, but it is also involved in transcription-independent apoptosis [26], [27].

It has been reported that ROS are generated during the redox cycling of β-lap [1]. In the present study, we examined the role of ROS in the cell death caused by combined treatment with IR and β-lap. We observed that both the rate and magnitude of ROS generation were markedly greater (Fig. 2A and B), and thus apoptosis was greater after combined treatment, compared to treatment with IR or β-lap alone in NQO1^+^-MDA-MB-231 cells (Fig. 2C).

We demonstrated that the rapid and strong activation of ERK induced by combined treatment with IR and β-lap is critically associated with the induction of apoptotic cell death (Fig. 3). Consistent with previous reports [28]–[30], we found that β-lap in combination with IR causes a rapid activation of JNK in NQO1^+^-MDA-MB-231 cells (Fig. 3A). However, activation of p38 MAPK did not appear to be involved in response to the combined treatment in NQO1^+^-MDA-MB-231 cells (Fig. 3A). In this context, inhibition of p38 MAPK by pretreating with SB203580 did not alter combined treatment-induced apoptotic cell death in NQO1^+^-MDA-MB-231 cells (Fig. 3B). The JNK activation caused by combined treatment was first evident within 0.5 h and decreased in 2 h in NQO1^+^-MDA-MB-231 cells (Fig. 3A). This reduction of JNK activity after 2 h may be due to the action of phosphatases, such as MAPK phosphatase 2 (MKP2) and -7 (MKP7), which have been reported to decrease the activity of intracellular MAPK signaling pathways [31]–[33]. ERK has been reported to increase the expression and activity of MKP2 and MKP7, leading to suppression of JNK [31], [33]. Similarly, because our results indicated that ERK activity was elevated for 3 h after combined treatment in NQO1^+^-MDA-MB-231 cells (Fig. 3A), ERK seems to participate in inducing the expression and activity of MKP2 or MKP7 for inhibition of JNK activity.

In this study, pretreatment of cells with NAC completely inhibited combined treatment-induced activation of ERK and JNK, and subsequent apoptotic cell death, demonstrating that increased intracellular ROS generation is critical for the activation of ERK and JNK (Fig. 4A). Increase of intracellular ROS has been reported to activate the Ras/Raf/ERK pathway by stimulating receptor tyrosine kinases or directly oxidizing the cysteine residues of Src, Ras or Raf [34]. Furthermore, several recent reports have indicated that ASK1, a MAPKKK for JNK activation, forms a complex with reduced thioredoxin (Trx) in non-stressed cells [35], [36]. Oxidation of Trx by ROS releases ASK1 from the complex and leads to JNK activation, possibly through dimerization of ASK1 [35], [36]. Therefore, further studies are required to clarify the role of ASK1 in the JNK activation that occurs in response to combined treatment with IR and β-lap.

Interestingly, inhibition of ERK by a targeted siRNA substantially suppressed combination treatment-induced ROS generation, whereas siRNA-mediated JNK knockdown did not (Fig. 4B). These results demonstrate that activation of ERK by combined treatment substantially contributes to ROS generation, and indicates the existence of positive feedback regulation between ERK activation and ROS generation. Several reports have demonstrated that ERK activation induces phosphorylation of a serine residue in p47 phox, a subunit of NADPH oxidase, thereby promoting the translocation of p47 phox to the cellular membrane, a perquisite for binding to and activation of the NADPH oxidase responsible for ROS generation [37], [38]. Pretreatment of NQO1^+^-MDA-MB-231 cells with DPI (diphenylene iodonium), a pharmacological inhibitor of NADPH oxidase, prior to combined treatment with IR and β-lap, significantly inhibited the ROS generation and apoptotic cell death caused by combined treatment (data not shown). Further studies are needed to elucidate the possible role of ERK in the generation of ROS generation in response to combined treatment with IR and β-lap.

In agreement with our previous report [39], β-lap alone caused a slight elevation of ER stress and the induction of ER stress by combined treatment with IR and β-lap was far more evident than by β-lap alone (Fig. 5A). Consistent with previous reports that ROS disrupt ER homeostasis [40]–[42], we found that inhibition of ROS generation by pretreatment with NAC significantly suppresses the induction of ER stress induced by combined treatment with IR and β-lap (Fig. 5D). However, Sal, an ER stress inhibitor, did not affect ROS generation (Fig. 5C), but effectively blocked apoptotic cell death (Fig. 5B), indicating that the induction of ER stress is mainly due to ROS generation. It is well established that ER stress activates JNK [43]–[46]. In agreement with previous reports, the suppression of ER stress by Sal effectively blocked JNK activation (Fig. 5E) and subsequent apoptotic cell death induced by combined treatment with IR and β-lap (Fig. 5B), but as mentioned above, it did not inhibit ROS generation (Fig. 5C). Furthermore, inhibition of JNK by pretreatment with SP600125 did not attenuate combined treatment-induced ER stress (Fig. 4C), indicating that JNK activation acts downstream of ER stress to induce apoptotic cell death. These results further support our conclusion that the induction of ER stress by ROS plays a crucial role in JNK activation and subsequent apoptotic cell death in response to combined treatment with IR and β-lap. Sal is known to selectively block ER stress-induced apoptotic cell death by inhibiting the dephosphorylation of eIF2α [47], [48]. However, it is unclear how dephosphorylation of eIF2α by Sal suppressed JNK activation, as we observed in our study. Additional studies are needed to clarify the inhibitory effect of Sal on the activation of JNK by ER stress in response to combined treatment with IR and β-lap.

Caspase activation was reported to be involved in the β-lap-induced apoptotic cell death of breast cancer cells [2]. Furthermore, although caspase-3 was well known to be the downstream effector of activated caspase-8 in death receptor-mediated cell death pathway [14], we observed that the activations of caspase-9 and -3 preceded that of caspase-8 after the combined treatment with IR and β-lap (Fig. 6A). Several reports also indicated that caspase-3 can cleave pro-casapsae-8 *in vitro* [49], and proposed the possibility that activation of caspase-8 in several anticancer drug treatments or disease may be mediated by caspase-9 or -3 [50]–[54]. In addition, combined treatment with IR and β-lap also caused the release of cytochrome *c*, expected to initiate activation of caspase-9 (Fig. 6B). Therefore, in agreement with these reports, caspase-9 activation via mitochondrial pathways may play a key role in activation of caspase-8 by combined treatment with IR and β-lap. However, interestingly, inhibition of caspases failed to inhibit apoptosis indicating activation of caspases is not a major or a solo mechanism for the induction of apoptosis by combined treatment with IR and β-lap (Fig. 6D).

AIF is known to function in a caspase-independent apoptosis pathway. Mitochondrial AIF is translocated to the nucleus in response to death stimuli, where it initiates nuclear condensation and DNA fragmentation [7], [55]. Consistent with these findings, we observed that AIF was translocated to the nucleus after combined treatment with IR and β-lap (Fig. 6E), and demonstrated that AIF activity was required for combined treatment-induced apoptosis (Fig. 6F). Proteolytic cleavage of native Bax (21 kDa) into an 18 kDa form by calpain has been shown to occur in cancer cells treated with a variety of chemotherapeutic drugs [15], [17]. This cleaved form of Bax is more potent than the native form in terms of disruption of mitochondrial transmembrane potential, release of cytochrome c and AIF from the mitochondria, and subsequent induction of apoptotic cell death [15], [17], [56]. We found that combined treatment with IR and β-lap induced Bax cleavage without altering Bcl-2 protein levels (Fig. 7A). We also observed that the cleaved form of Bax was translocated to mitochondria upon combined treatment (Fig. 7C), coinciding with the observed changes in mitochondrial transmembrane potential and nuclear translocation of AIF (Fig. 7B).

In conclusion, we demonstrate that, compared to individual treatment with IR or β-lap, β-lap in combination with IR was markedly more potent in causing apoptotic cell death. Combined treatment induced mitochondrial apoptotic cell death in an NQO1-dependent manner through positive feedback regulation between ERK and ROS, which contributed to the induction of ER stress and led to JNK activation and nuclear translocation of AIF (Fig. 7H). Our results demonstrate that β-lap may effectively improve the therapeutic efficacy of radiation therapy by targeting NQO1.

## Materials and Methods

### Reagents

β-lap was purchased from Biomol (Plymouth Meeting, PA), and was dissolved in DMSO. Antibodies against p38 MAPK, JNK2, JNK1/2, ERK1/2, Bax, Bcl2, AIF and Nucleoporin p62 were purchased from Santa Cruz Biotechnology, Inc. (Santa Cruz, CA). An antibody against mitochondrial heat shock protein 70 (HSP70) was obtained from Affinity Bioreagents (Golden, CO). Anti-β-actin, -rabbit IgG, -mouse IgG and N-acetyl-L-cysteine (NAC) were purchased from Sigma (St. Lous, MO). Antibodies against phospho-p38 MAPK (Thr180/Tyr182), phospho-JNK1/2 (Thr183/Tyr185), phospho-ERK1/2 (Thr202/Tyr204), phospho-eIF2α (Ser51), eIF2α and active caspase-3 were obtained from Cell Signaling Technology (Beverly, MA). Inhibitors specific to JNK (SP600125), MEK/ERK (PD98059), p38 MAPK (SB203580), caspases (z-VAD-fmk), protease (MG132) and ER stress (Salubrinal) were purchased from Calbiochem (San Diego, CA).

### Cell culture

Parental NQO1^−^-MDA-MB-231 human breast cancer cells, which are NQO1-deficient, and NQO1^+^-MDA-MB-231 human breast cancer cells, which are stably transfected with NQO1, were obtained from Dr. David Boothman (University of Texas Southwestern Medical Center, Dallas, TX). Cells were cultured in RPMI 1640 medium (Gibco BRL, Grand Island, NY) supplemented with 10% (v/v) bovine calf serum (Gibco BRL), penicillin (50 units/ml), and streptomycin (50 µg/ml), in a 37°C incubator under a mixture of 95% air and 5% CO_2_.

### Irradiation

Cells were exposed to γ-rays with a 137Cs irradiation source (Model 68; J.L. Shepherd and Associates, Glenwood, CA) at a dose rate of 200–300 cGy/min.

### Transfection of small interfering RNA

RNA interference with small interfering RNAs (siRNAs) was carried out using double-stranded RNA molecules. ERK1/2 siRNA (#6560) was purchased from Cell Signaling Technology (Beverly, MA). siRNAs against AIF (5′-GCA AGU UAC UUA UCA AGC UTT-3′) and JNK2 (5′-CUG UAA CUG UUG AGA UGU ATT-3′) were purchased from Bioneer Corporation (Daejeon, Korea). An unrelated control siRNA (5′-CCA CTA CCT GAG CAC CCA G-3′) that targets the green fluorescent protein DNA sequence was used as a control. For transfection, cells were seeded on 60-mm dishes and transfected at 30% confluency with siRNA duplexes (100 nM) using Lipofectamine 2000 (Invitrogen, Carlsbad, CA) in accordance with the manufacturer's instructions. Assays were performed 24 h after transfection.

### Quantification of clonogenic death

Various numbers of cells were plated on 60-mm dishes and treated with 2 µM β-lap, a range of doses of IR (0∼4 Gy) or β-lap (2 µM) in combination with and IR (0∼4 Gy). Cells were then incubated for 14 days at 37°C in 5% CO_2_ incubator to allow colonies to form. Prior to counting colonies, the culture medium was decanted, and the cells were fixed in 95% methanol, stained with 0.5% crystal violet, and the numbers of colonies (>50 cells/colony) from triplicate dishes were counted. Mean colony numbers were plotted relative to those formed by unirradiated cells.

### Quantification of apoptosis

Cells were collected by trypsinization, washed 2 times with phosphate-buffered saline (PBS); resuspended in 1 ml PBS containing 0.1% Triton X-100, 0.1 mM EDTA, 10 mg/ml DNase-free RNase A, and 2 mg/ml propidium iodide (PI); and incubated for 1 h in the dark at 37°C. Apoptotic cells were detected by flow cytometry using a FACSCalibur system (Becton Dickinson, San Jose, CA). Apoptosis was measured as the percentage of cells in the sub-G1 population.

### Measurement of ROS generation

Cells were incubated at 37°C for 30 min in 10 µM 2′,7′-dichlorofluorescein-diacetate (DCFH-DA; Molecular Probes, Eugene, OR) or 4 µM dihydroethidium (DHE; Molecular Probes, Eugene, OR), harvested by trypsinization, and washed three times with cold PBS. ROS levels were determined by flow cytometry, as described previously [7].

### Western blot analysis

Cells were treated with lysis buffer [40 mmol/L Tris-Cl (pH 8.0), 120 mmol/L NaCl, and 0.1% NP40] supplemented with protease inhibitors, then centrifuged for 15 min at 12,000× g. Proteins were separated by sodium dodecyl sulfate-polyacrylamide gel electrophoresis (SDS-PAGE) and transferred to nitrocellulose membranes (Bio-Rad, Hercules, CA). The membranes were blocked with 5% nonfat dry milk in Tris-buffered saline and subsequently incubated for 1 h with primary antibodies at room temperature. Blots were developed with a peroxidase-conjugated secondary antibody, and immunoreactive proteins were visualized using enhanced chemiluminescence reagents (Amersham Biosciences, Piscataway, NJ), as recommended by the manufacturer. β-actin, Nucleoporin p62 and mitochondrial HSP 70 were used as a loading control, a nuclear marker, and a mitochondrial marker, respectively.

### Measurement of mitochondrial membrane potential

Cells were incubated for 30 min in 30 nM 3,3′-dihexyloxacarbocyanine iodide (DiOC6(3); Molecular Probes, Eugene, OR) at 37°C, harvested by trypsinization, and washed three times with cold PBS. Mitochondrial membrane potential was determined by flow cytometry, as described previously [7].

### Preparation of mitochondrial and nuclear fractions

Cells were collected and washed twice in ice-cold PBS, resuspended in isotonic homogenization buffer [250 mM sucrose, 10 mM KCl, 1.5 mM MgCl2, 1 mM Na-EDTA, 1 mM dithiothreitol, 0.1 mM phenylmethylsulfonylfluoride, 10 mM Tris-HCl (pH 7.4)], incubated on ice for 20 min, and homogenized using a Dounce glass homogenizer (70 strokes) fitted with a loose pestle (Wheaton, Millville, NJ). Cell homogenates were spun at 30× g to remove any unbroken cells. Supernatants were then respun for 10 min at 750× g to separate nuclear and mitochondrial fractions. Each pellet (nuclear fraction) was washed three times with homogenization buffer, and resuspended in nuclear lysis buffer [50 mM Tris-HCl (pH 7.5), 150 mM NaCl, 1% (v/v) NP40, 0.5% (w/v) sodium deoxycholate] containing protease inhibitors, prior to Western blot analysis. After pelleting the nuclear fraction, the supernatant was further subjected to 30 min of centrifugation at 14,000× g to obtain a mitochondria-rich fraction. Pellets were washed once with homogenization buffer and then resuspended in mitochondrial lysis buffer [150 mM NaCl, 50 mM Tris-HCl (pH 7.5), 1% (v/v) NP40, 0.25% (w/v) sodium deoxycholate, and 1 mM EGTA] containing protease inhibitors prior to Western blot analysis.

### Preparation of cytosolic fractions for cytochrome *c* measurement

Cells were collected and washed twice in ice-cold PBS, resuspended in isotonic homogenization buffer [250 mM sucrose, 10 mM KCl, 1.5 mM MgCl2, 1 mM Na-EDTA, 1 mM dithiothreitol, 0.1 mM phenylmethylsulfonylfluoride, 10 mM Tris-HCl (pH 7.4)], incubated on ice for 20 min, and homogenized using a Dounce glass homogenizer (70 strokes) fitted with a loose pestle (Wheaton, Millville, NJ). Cell homogenates were spun at 1,000× g to remove unbroken cells, nuclei, and heavy membranes. The supernatant was re-spun at 14,000× g for 30 min to collect the cytosolic (the supernatant) fractions.

### Statistical analysis

All data presented are representative of at least three separate experiments. Comparisons between groups were analyzed using Student's t-test (SPSS Statistics version 17.0, Chicago, IL). p values<0.05 (indicated by * on figures) were considered to be significant.
